# A two-leaf daily GPP model based on a rectangular hyperbolic model adjusted for air temperature and vegetation type

**DOI:** 10.3389/fpls.2025.1555482

**Published:** 2025-03-11

**Authors:** Qiuxiang Yi, Fumin Wang

**Affiliations:** ^1^ Zhejiang University of Water Resources and Electric Power, School of Geomatics, Hangzhou, China; ^2^ Zhejiang Key Laboratory of Agricultural Remote Sensing and Information Technology, Zhejiang University, Hangzhou, China; ^3^ Institute of Applied Remote Sensing and Information Technology, Zhejiang University, Hangzhou, China; ^4^ Ministry of Education Key Laboratory of Environmental Remediation and Ecological Health, Zhejiang University, Hangzhou, China

**Keywords:** gross primary productivity, two-leaf, modeling, rectangular hyperbolic model, light use efficiency, enzyme kinetic model

## Abstract

An accurate and easy-to-use gross primary productivity (GPP) model is essential for studying the spatial and temporal dynamics of the terrestrial carbon cycle on a global scale. Light use efficiency (LUE) models and process-based models are the two most commonly used approaches for GPP modeling. While LUE models are simpler and more user-friendly, process-based models often achieve higher accuracy due to their detailed structure. In this study, we introduce a new two-leaf GPP model (TL-RHM) with two expression forms at a daily temporal resolution. The TL-RHM is developed by temporally integrating a modified rectangular hyperbolic model that incorporates the effects of temperature variations on GPP across various vegetation types. The performance of the TL-RHM is evaluated using data from 21 CO_2_ eddy-covariance flux sites, covering four vegetation types: evergreen needleleaf forest, deciduous broadleaf forest, grassland, and evergreen broadleaf forest. The results demonstrate that the daily GPP simulated by the TL-RHM agrees well with the measured GPP for both calibration and validation datasets across all four vegetation types. These findings highlight the potential of the TL-RHM to accurately simulate daily GPP with a relatively simple model structure, offering a valuable tool for long time-series GPP simulations at regional or global scales.

## 1 Introduction

Gross primary productivity (GPP) is a key component of the terrestrial carbon cycle (Sarkar et al., 2024; Marsh et al., 2025; Cheng et al., 2022; Mäkelä et al., 2008), and it is usually modeled using light use efficiency (LUE) models and process-based models at time steps of ranging from half hour, an hour, a day, 8 days, and a month. LUE models are commonly used for time steps of a day, 8 days, and a month (Law and Waring, 1994; Running et al., 2004; Yuan et al., 2014). These models have the advantages of simple model structure and high computation efficiency, which can be combined with remotely sensed data to perform large-scale GPP modeling. However, LUE models are insufficient in mechanism descriptions of biophysical and biological processes involved in photosynthesis because they are highly integrated in terms of time and space. From the perspective of time, these LUE models do not consider the effects of diurnal variations of meteorological variables to photosynthesis, and also they do not consider the effects of day-to-day variations of meteorological variables when multiday composites of GPP are computed. From the perspective of space, they are usually based on the big-leaf model, which does not separate canopy leaves into sunlit and shaded leaves. So these spatiotemporal characteristics of LUE models result in their limitations in GPP computation, especially for daily GPP modeling (Raczka et al., 2013; Wang et al., 2013).

Process-based models (PBMs) are considered to have the ability for accurate simulation of GPP. Most of them are based on Farquhar’s enzyme kinetic (EK) photosynthesis model (Farquhar et al., 1980) or its variants such as Baldocchi’s model (Baldocchi, 1994), which couples Farquhar’s model with the Ball–Woodrow–Berry (BWB) stomatal conductance model (Ball et al., 1987) to derive an instantaneous leaf-scale photosynthesis model. Although both Farquhar’s and Baldocchi’s models are instantaneous models, they are never run at a second time step but are often run at half-hourly or hourly time steps (Wang et al., 2001; Ju et al., 2006). In each step, the half-hourly or hourly averaged values of meteorological variables are inputted for GPP computation. The advantage of PBMs is that they are developed based on the mechanism of biophysical and biological processes. Therefore, simulation accuracies of GPP by PBMs are considered to be better than those by LUE models. However, the use of PBMs may be limited in large-scale and long time-series GPP simulations with high spatial resolution due to the complex model structures and small time steps. This is because measured half-hourly or hourly input data are unavailable at a global scale and interpolated data may result in some uncertainties.

In fact, there is another type of GPP model, the rectangular hyperbolic model (RHM), which is usually applied in temperature-controlled experiments (Pachepsky et al., 1996; Saito et al., 2005). Compared to the two groups of GPP models mentioned above, RHM is rarely used for long-term large-scale GPP modeling because its two parameters—quantum yield and maximum photosynthetic rate—vary temporally for different vegetation types. The use of fixed parameters of RHM under variable natural conditions can lead to errors in GPP calculations.

Above all, the commonly used models have some shortcomings in efficient and accurate GPP computation. But when studying the global terrestrial ecosystem carbon cycle, we often need to calculate GPP at large scales (regional or global), over long time series, and with high spatial resolution. Therefore, we urgently need an easy-to-use and efficient GPP model with high simulation accuracy. One possible way to solve the problem is temporal integration of instantaneous Baldocchi’s photosynthesis model with respect to time and space as Baldocchi’s model is based on Farquhar’s photosynthesis model and takes only meteorological data as inputs. The integration can be performed because most meteorological variables follow predictable diurnal courses over the course of a day. For example, radiation usually follows a sine function with a peak at solar noon (Kimball and Bellamy, 1986). However, an analytical solution for the integration of Baldocchi’s model with respect to time is difficult to accomplish due to the complexity of the equations (Wang et al., 2014a). In order to overcome this problem, we present an alternative way to integrate Baldocchi’s model by establishing a relationship between Baldocchi’s model and the rectangular hyperbolic model. First, we develop a temperature- and vegetation type-adapted rectangular hyperbolic model by linking it to Baldocchi’s photosynthesis model with high fidelity. Then, we integrate the modified rectangular hyperbolic model with respect to time to obtain a leaf-scale daily GPP model. Finally, we couple the daily GPP model with a two-leaf upscaling strategy to get a two-leaf daily GPP model with two expression forms, namely, TL-RHM_sine and TL-RHM_sinesine. Therefore, the objective of this study is to develop a new daily canopy-scale model for accurate and efficient GPP calculation. The model is of both photosynthesis mechanism and simple model structure, and it is expected to provide support for large-scale, long time-series, and high spatial resolution GPP simulation.

## 2 Materials and methods

### 2.1 Flux tower sites and data

The flux tower datasets used to calibrate and validate the new proposed model are acquired from the AmeriFlux website (http://ameriflux.ornl.gov) and the Canadian Carbon Program (CCP) website (http://fluxnet.ccrp.ec.gc.ca). Twenty-one sites covering evergreen needle leaf forest (ENF), deciduous broadleaf forest (DBF), grassland, and evergreen broadleaf forest (EBF) are selected. For each site, half-hourly global radiation or photosynthetically active radiation, relative humidity, air temperature, and CO_2_ flux data, as well as some key datasets such as max LAI, can be used in the study. The half-hourly data associated with equipment failures have been gap-filled using the artificial neural network method (Papale and Valentini, 2003) or Barr’s gap-filling method (Barr et al., 2004). In order to obtain the inputs of a new daily model, the half-hourly measurements of radiation and GPP are summed to daily values, while the half-hourly measurements of air temperature are used to extract the maximum and minimum air temperatures during a day. Since the daily GPP is simulated for a whole year, the time-series LAI data are needed for time-series GPP modeling. For most of the sites, there are no measured time-series LAI data, but the measured maximum LAI data are available. So, the measured maximum LAI data are combined with the time-series MODIS LAI to obtain time-series LAI data as the model input. To evaluate the model performance with independent data, for each of the vegetation types, nearly half of the sites are used for model calibration, and the remaining sites are used for model validation (**Table 1**).

**Table 1:** The description of sites used in this study.

Vegetation type	Site ID	Longitude	Latitude	Year	Category	References
Evergreen needleleaf forest	US_NR1	−105.546	40.033	2005	Calibration	Monson et al. (2005)
	US_Ho1	−68.740	45.204	2004	Calibration	Hollinger et al. (2004)
	US_Wrc	−121.952	45.821	2004	Calibration	Falk et al. (2008)
	Ca_DF49	−125.335	49.869	2008	Validation	Jassal et al. (2009)
	US_Ho2	−68.747	45.209	2004	Validation	Richardson and Hollinger (2005)
	US_SP2_	−82.2448	29.765	2004	Validation	Zhao et al. (2013)
Deciduous broadleaf forest	US_MOz	−92.200	38.744	2007	Calibration	Gu et al. (2007)
	US_UMB	−84.714	45.560	2004	Calibration	Curtis et al. (2002)
	Ca_OA	−106.198	53.629	2008	Calibration	Barr et al. (2004)
	US_Bar	−71.288	44.065	2006	Validation	Jenkins et al. (2007)
	US_Ha1_	−72.172	42.538	2004	Validation	Urbanski et al. (2007)
	US_MMS	−86.413	39.323	2004	Validation	Schmid et al. (2000)
	US_WCr	−90.080	45.806	2004	Validation	Cook et al. (2004)
Grassland	US_Var	−120.951	38.407	2005	Calibration	Baldocchi (2003)
	US_Fwf	−111.772	35.445	2007	Calibration	Dore et al. (2010)
	US_IB2	−88.241	41.841	2006	Calibration	Allison et al. (2005)
	US_Dk1	−79.093	35.971	2001	Validation	Oren et al. (2006)
	US_Aud	−110.509	31.591	2005	Validation	Krishnan et al. (2012)
	US_Goo	−89.874	34.255	2004	Validation	Wilson and Meyers (2007)
Evergreen broadleaf forest	BR_Sa1	−54.959	−2.857	2004	Calibration	Wick et al. (2005)
	BR_Sa3	−54.9714	−3.018	2001	Calibration	Goulden et al. (2006)
	BR_Sa3	−54.9714	−3.018	2002	Validation	Goulden et al. (2006)

### 2.2 A leaf-scale temperature and vegetation type-adapted rectangular hyperbolic model

The traditional RHM can be expressed as Equation 1:


(1) $$GCA_{leaf}(t)=\frac{\alpha P_{m}APAR(t)}{P_{m}+\alpha APAR(t)}$$

where *GCA*_leaf_ is the leaf photosynthetic rate at time *t*, and *α* and *P*_m_ are the quantum yield and maximum photosynthetic rate at light saturation conditions, respectively.

The traditional RHM is usually applied in temperature-controlled experiments or over a few days during which temperature is assumed to be constant (Wang et al., 2014b). In our study, we improve the traditional RHM by establishing a relationship with Baldocchi’s photosynthesis model to obtain a modified RHM as follows: 1) for a given temperature and vegetation type, leaf-scale GPPs are modeled by Baldocchi’s photosynthesis model with a series of photosynthetic photon flux density (*PPFD*) values ranging from 50 μmol m^−2^ s^−1^ to 2,000 μmol m^−2^ s^−1^ as inputs; 2) the two parameters, *α* and *P*_m_, are obtained by regressing *PPFD* against GPP using the rectangular hyperbolic model with fitted *R* ^2^ close to 1 (**Supplementary Figure S1**); and 3) the above two steps are repeated for all combinations of temperature from 1°C to 40°C and the maximum rate of Rubisco-mediated carboxylation (*V*_cmax,25_) from 20 μmol m^−2^ s^−1^ to 180 μmol m^−2^ s^−1^ to get the *α* and *P*_m_ distribution maps (Ceccato et al., 2002) (see **Supplementary Figures S2, S3**). It should be pointed out that, for all combinations of temperature and *V*_cmax,25_, the *R* ^2^ values of all fitted RHMs are greater than 0.99, meaning that, for a vegetation type, the modified RHM can perform as well as Baldocchi’s model under fixed temperature conditions (Wang et al., 2014a). The fact that RHM can accurately simulate the photosynthetic process under fixed temperature conditions has been proven in a previous study (Pachepsky et al., 1996). Therefore, we developed a leaf-scale RHM that is adjusted for air temperature and vegetation type, which is expressed as Equation 2:


(2) $$GCA_{leaf}(V_{\text{cmax},25},T,t)=\frac{\alpha (V_{\text{cmax},25},T)\times P_{m}(V_{\text{cmax},25},T)\times APAR(t)}{P_{m}(V_{\text{cmax},25},T)+\alpha (V_{\text{cmax},25},T)\times APAR(t)}$$

where *GCA*_leaf_ is the leaf-scale photosynthetic rate for a vegetation type with a fixed *V*_cmax,25_ at a temperature *T*. *α* and *P_m_* are the two parameters of *GCA*_leaf_, and they are determined by temperature *T* and *V*_cmax,25_.

### 2.3 Integration of the modified RHM with respect to time

The daily leaf-scale GCA can be calculated as:


(3) $$GCA_{daily,leaf}(V_{\text{cmax},25},T)=\int_{t_{rise}}^{t_{set}}\frac{\alpha (V_{\text{cmax},25},T_{p})\times P_{m}(V_{\text{cmax},25},T_{p})\times APAR(t)}{P_{m}(V_{\text{cmax},25},T_{p})+\alpha (V_{\text{cmax},25},T_{p})\times APAR(t)}$$

where *t*_rise_ and *t*_set_ are the times of sunrise and sunset, respectively. *T_p_* is the average temperature during the active photosynthesis period in the day.

The diurnal variation of *APAR* is described using a simple sine function (Kimball and Bellamy, 1986) or a squared sine function:


(4) $$APAR_{t}=APAR_{\text{noon}}\sin\left[\frac{\text{π(}t\text{ - }t_{\text{rise}})}{t_{\text{set}}-t_{\text{rise}}}\right]\text{ = }\frac{\text{π}APAR_{daily}}{2\text{Daylength}}\sin\left[\frac{\text{π(}t\text{ - }t_{\text{rise}})}{t_{\text{set}}-t_{\text{rise}}}\right]$$

(5) $$APAR_{t}=APAR_{\text{noon}}\sin^{2}\left[\frac{\text{π(}t\text{ - }t_{\text{rise}})}{t_{\text{set}}-t_{\text{rise}}}\right]\text{ = }\frac{2APAR_{daily}}{\text{Daylength}}\sin^{2}\left[\frac{\text{π(}t\text{ - }t_{\text{rise}})}{t_{\text{set}}-t_{\text{rise}}}\right]$$

Therefore, daily leaf-scale GPPs are expressed in the forms of Equations 6, 7 by substituting *APAR* in Equation 3 with Equation 4 or Equation 5. From Equation 4, we obtain the daily model (TL-RHM_sine).


(6) $$GPP_{daily}=\left\{ \begin{array}{l} P_{m}\text{Daylength}\left[1-\frac{2a}{\pi \sqrt{a^{2}-1}}\left(\frac{\pi }{2}-\arctan\left(\frac{1}{\sqrt{a^{2}-1}}\right)\right)\right]\;a^{2}>1\\ P_{m}\text{Daylength}\left(1-\frac{\pi }{2}\right)\;a^{2}=1\\ P_{m}\text{Daylength}\left[1-\frac{a}{\pi \sqrt{a^{2}-1}}\ln\left(\frac{1+\sqrt{1-a^{2}}}{1-\sqrt{1-a^{2}}}\right)\right]\;a^{2}<1\; \end{array}\right.$$

where Daylength is the length of day, $a=\frac{P_{m}}{\alpha APAR_{noon}}$ , $APAR_{noon}=\frac{\pi APAR_{daily}}{2\text{Daylength}}$ And from Equation 5, we obtain the daily model (TL-RHM_sinesine):


(7) $$GPP_{daily}=P_{m}\text{Daylength}\left(1-\sqrt{\frac{a}{a+1}}\right)\;$$

where Daylength is the length of day, $a=\frac{P_{m}}{\alpha APAR_{noon}}$ , $APAR_{noon}=\frac{2APAR_{daily}}{\text{Daylength}}$

### 2.4 Upscaling leaf-scale GPP to canopy-scale using a two-leaf strategy

The canopy-scale GPP (*GPP*_canopy_) can be calculated as the sum of the total GPP of sunlit and shaded leaves (Chen et al., 1999, 2012) (Equation 8):


(8) $$GPP_{\text{canopy}}\;=\;GCA_{\text{sunlit}}\times LAI_{\text{sunlit}}+GCA_{\text{shaded}}\times LAI_{\text{shaded}}$$

where the subscripts “sunlit” and “shaded” denote the sunlit and shaded components of *GCA* and leaf area index (*LAI*). The *GCA*_sunlit_ and *GCA*_shaded_ are calculated using Equations 6, 7. Moreover, the APAR of sunlit and shaded leaves and the total LAI separation into sunlit and shaded LAI are calculated using the methods of Chen et al. (1999). Therefore, the final daily canopy GPP models are the two two-leaf temperature and vegetation type-adapted rectangular hyperbolic models with the diurnal radiation following a sine function (Equation 4) and a squared sine function (Equation 5), called TL-RHM_sine and TL-RHM_sinesine, respectively. The inputs of the models are summarized in **Supplementary Table S1**.

Since the effect of relative humidity on GPP estimation is not considered in the new daily model, a scalar of vapor pressure deficit (*VPD*), *f*(*VPD*), is used to downregulate the daily GPP by TL-RHM under unfavorable conditions of high *VPD* (Equation 9), which will make the new daily model suitable for changeable environmental conditions.


(9) $$GPP_{\text{actual}}=GPP_{canopy}\times f(VPD)$$

where the *GPP*_actual_ is GPP regulated by *VPD*, and the *f*(*VPD*) can be expressed as follow (Equation 10):


(10) $$f(VPD)=\left\{ \begin{array}{l} 0\text{ VPD}\geq {\text{VPD}}_{\max}\\ \frac{VPD_{\max}-VPD}{VPD_{\max}-VPD_{\min}}{\text{ VPD}}_{\min}\leq \text{VPD}\leq {\text{VPD}}_{\max}\\ 1\text{ VPD}\leq {\text{VPD}}_{\min} \end{array}\right.$$

where *VPD*_max_, *VPD*_min_ are the parameters dependent on vegetation types (**Table 2**).

**Table 2:** Parameters calibrated for daily TL-RHM.

Parameters	Unit	Evergreen needleleaf forest	Deciduous broadleaf forest	Grassland	Evergreen broadleaf forest
*V*_cmax,25_	μmol m^−2^ s^−1^	52	63	95	30
*VPD*_max_	Pa	4,500	4,500	4,500	4,500
*VPD*_min_	Pa	650	650	650	650

## 3 Results

The CO_2_ eddy-covariance measurements from 21 sites with four vegetation types—ENF, DBF, grassland (GRA), and EBF (**Table 1**)—are used to evaluate the two-leaf rectangular hyperbolic model (TL-RHM) at daily temporal resolution. Since the two forms of TL-RHM correspond to different assumptions for diurnal radiation patterns, their performance in GPP simulations will also be evaluated with different vegetation types.

### 3.1 Calibration of the two-leaf rectangular hyperbolic model by measurements

**Figure 1:**
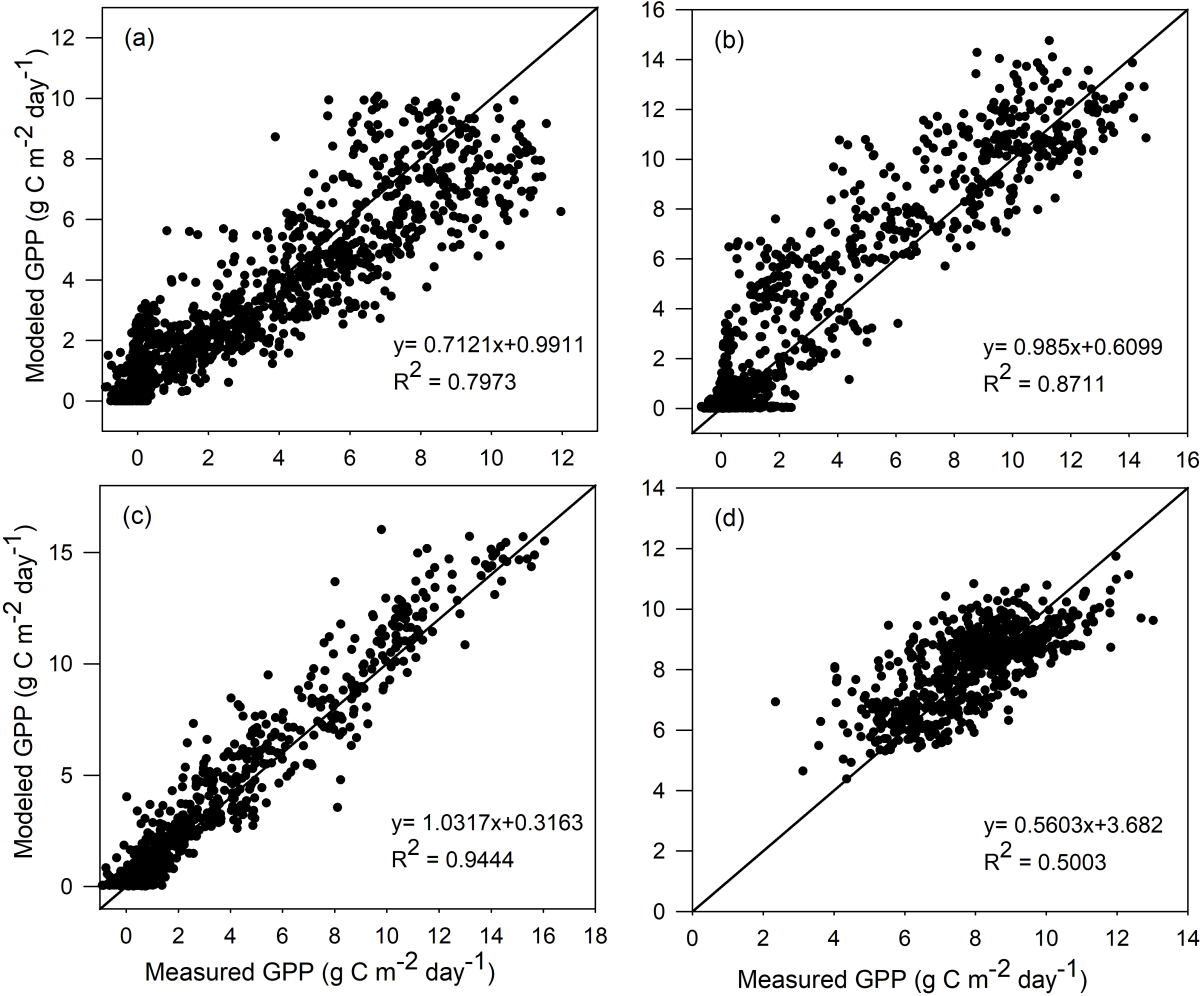
Scatterplots of GPP simulated by the TL-RHM_sine versus measured GPP for the calibration sites of **(a)** evergreen coniferous forest (three sites with 1,097 samples), **(b)** broadleaf deciduous forest (three sites with 1,097 samples), **(c)** grassland (three sites with 1,095 samples), and **(d)** evergreen broadleaf forest (two sites with 731 samples). Diagonal lines are the 1:1 lines.

**Figure 2:**
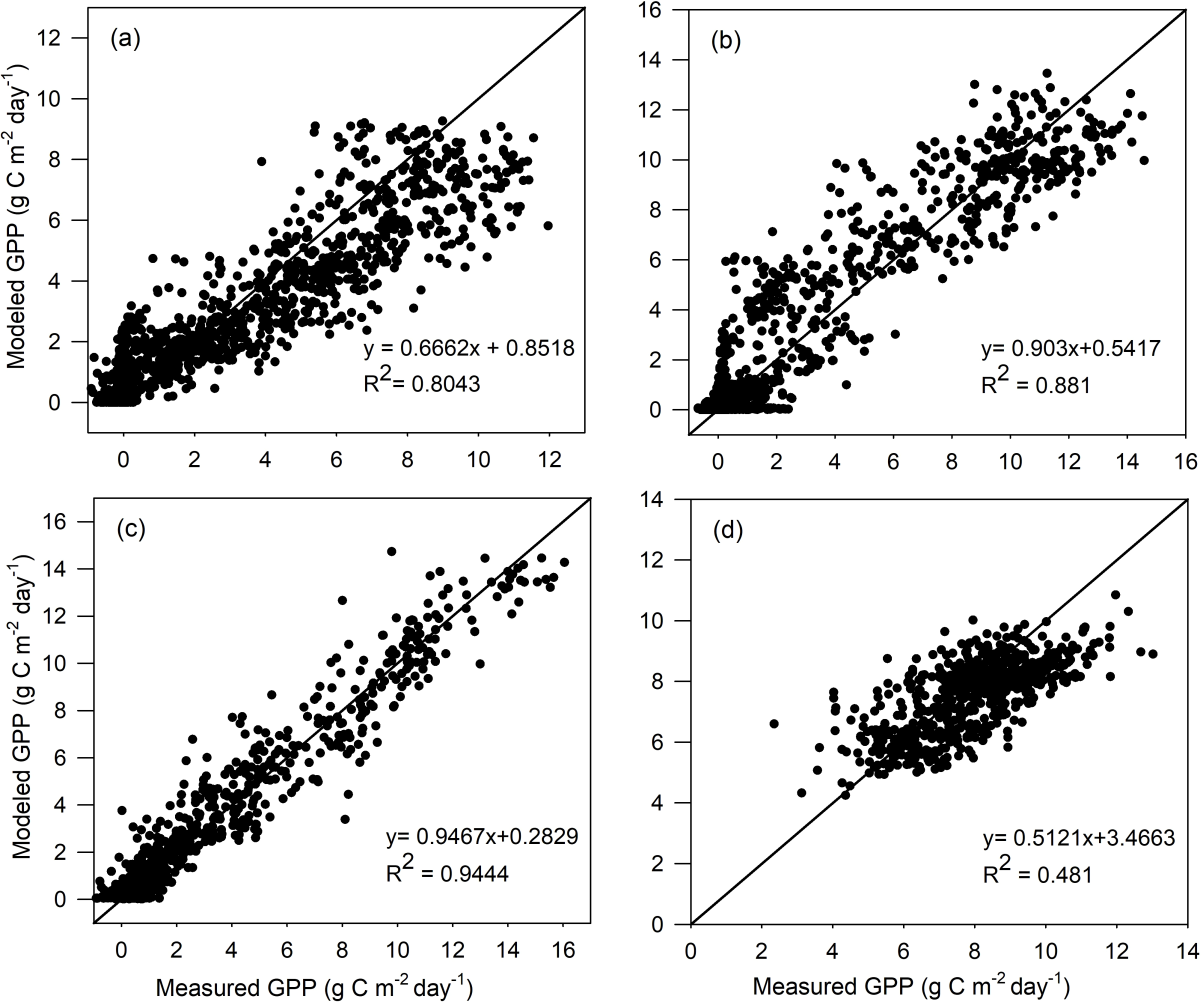
Scatterplots of GPP simulated by the TL-RHM_sinesine versus measured GPP for the calibration sites of **(A)** evergreen coniferous forest (three sites with 1,097 samples), **(B)** broadleaf deciduous forest (three sites with 1,097 samples), **(C)** grassland (three sites with 1,095 samples), and **(D)** evergreen broadleaf forest (two sites with 731 samples). Diagonal lines are the 1:1 lines.

The two-leaf rectangular hyperbolic model is calibrated by conducting ensemble runs in a range of *V*_cmax,25_, and then the optimal *V*_cmax,25_ is determined for each vegetation type. The calibration results are shown in **Table 2**. For the calibration dataset, the *R* ^2^ and RMSE between the modeled and measured GPP are computed for all four vegetation types, i.e., ENF, DBF, GRA, and EBF. As seen in **Figure 1**, the GPP values modeled by TL-RHM_sine are in good agreement with those of the measured GPP with *R* ^2^ values of 0.797, 0.871, and 0.944 for ENF, DBF, and GRA sites and with RMSE values of 1.619, 1.630, and 1.033 g C m^−2^ day^−1^ for ENF, DBF, and GRA sites, respectively. For the EBF site, the modeled and measured GPPs show a relatively poorer agreement with an *R* ^2^ value of 0.500. However, since the variation range of GPP is small for the tropical evergreen forest, TL-RHM performs acceptably well in tracing the seasonal variation of GPP with an RMSE of 1.212 g C m^−2^ day^−1^. The comparisons between the measured GPP and the modeled GPP by TL-RHM_sinesine follow a similar pattern for the four vegetation types with *R* ^2^ values of 0.804, 0.881, 0.944, and 0.481 (**Figure 2**) and RMSE values of 1.549, 1.465, 0.911, and 1.265 g C m^−2^ day^−1^ for the ENF, DBF, GRA, and EBF sites. From **Figures 1**, **2**, it can be seen that the TL-RHM_sine and TL-RHM_sinesine can accurately simulate daily variations of GPP for the ENF, DBF, and GRA sites, and both show a relatively poor performance for EBF sites due to their small variations of GPP. Although there is a small difference between TL-RHM_sine and TL-RHM_sinesine, TL-RHM_sinesine performs slightly better than TL-RHM_sine for ENF and DBF sites, while for EBF sites, the reverse is true.

For the calibration dataset, the modeled GPP by both TL-RHM_sine and TL-RHM_sinesine can track the seasonal variations of measured GPP for the four vegetation types. The modeled GPP also agrees well with day-to-day variations of measured daily GPP for most of the sites (**Supplementary Figures S4–S7**), but the two types of TL-RHM tend to overestimate GPP in the spring for the US_NR1 and US_Ho1 sites and are incapable of capturing the relatively high and low values of daily GPP for EBF sites.

### 3.2 Validation of the two-leaf rectangular hyperbolic model using measurements

**Figure 3:**
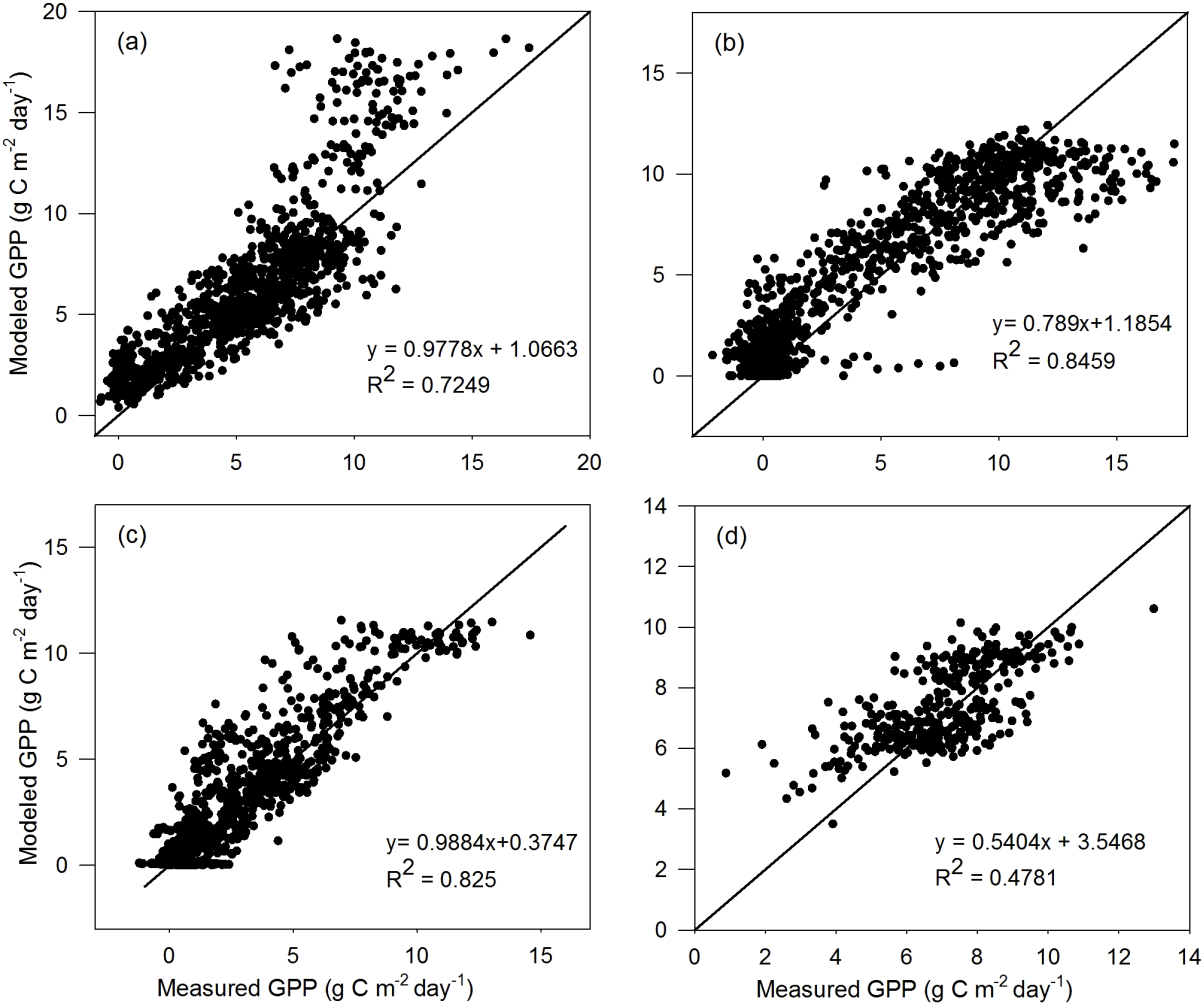
Scatterplots of GPP simulated by the TL-RHM_sine versus measured GPP for the validation sites of **(a)** evergreen coniferous forest (three sites with 1,098 samples), **(b)** broadleaf deciduous forest (four sites with 1,463 samples), **(c)** grassland (three sites with 1,096 samples), and **(d)** evergreen broadleaf forest (one site with 365 samples). Diagonal lines are the 1:1 lines.

**Figure 4:**
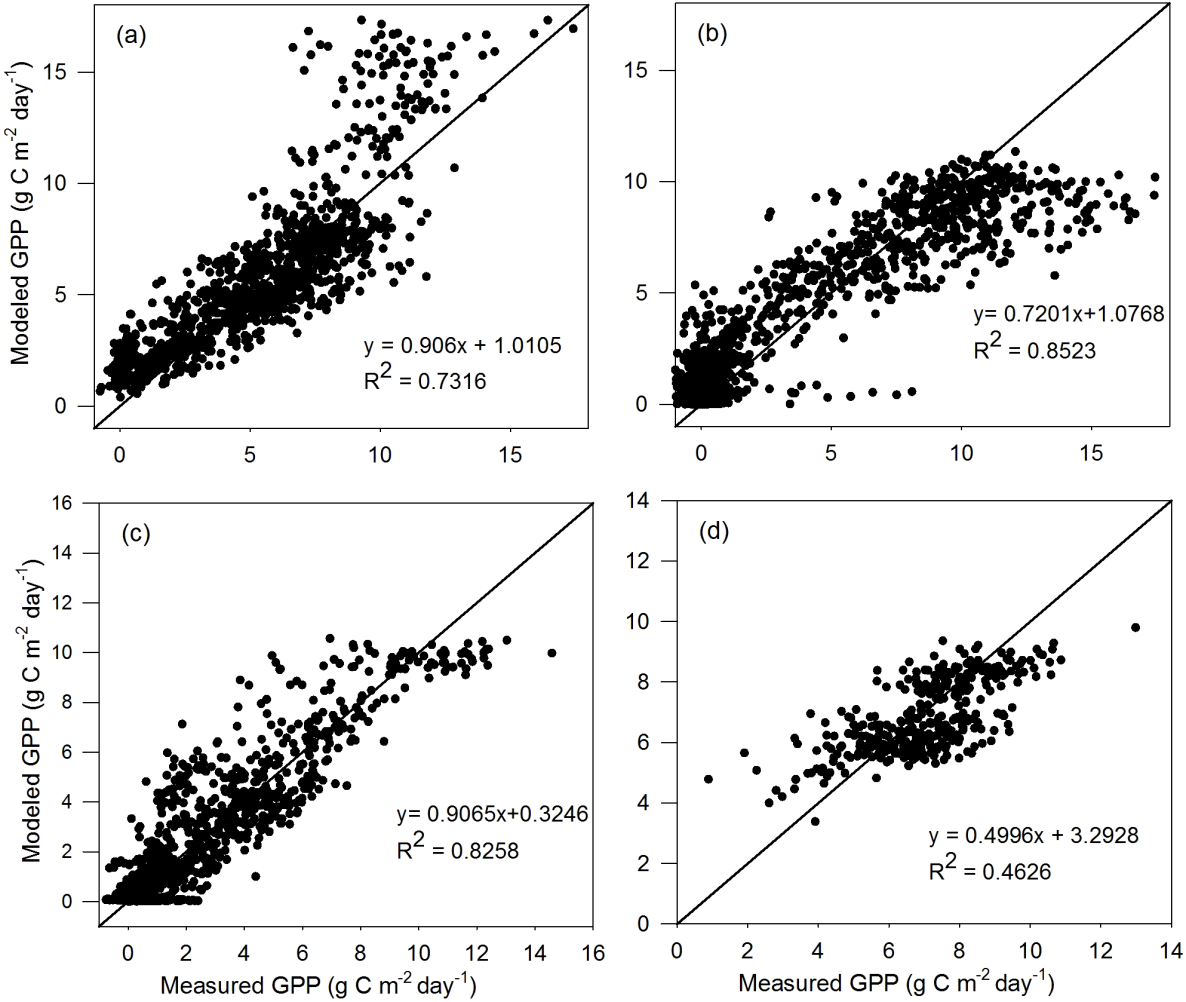
Scatterplots of GPP simulated by the TL-RHM_sinesine versus measured GPP for the validation sites of **(a)** evergreen coniferous forest (three sites with 1,098 samples), **(b)** broadleaf deciduous forest (four sites with 1,463 samples), **(c)** grassland (three sites with 1,096 samples), and **(d)** evergreen broadleaf forest (one site with 365 samples). Diagonal lines are the 1:1 lines.

The comparison results of the validation dataset are similar to those of the calibration dataset for the four vegetation types (**Figures 3**, **4**). For TL-RHM_sine, the relatively large *R* ^2^ values of 0.725, 0.846, and 0.825 are obtained with RMSE values of 2.261, 1.916, and 1.349 g C m^−2^ day^−1^ for the ENF, DBF, and GRA sites, respectively. The *R* ^2^ for EBF sites is relatively small, similar to that of the calibration dataset, with an RMSE of 1.172 g C m^−2^ day^−1^. For TL-RHM_sinesine, the *R* ^2^ values between the modeled and measured GPPs are 0.732, 0.852, 0.826, and 0.463 with RMSE values of 2.001, 1.854, 1.214, and 1.230 g C m^−2^ day^−1^ for the ENF, DBF, GRA, and EBF sites, respectively. In **Figures 3a**, **4a**, the points corresponding to the overestimation of GPP are mainly from Ca_DF49, that is because the *V*_cmax,25_ for that site is lower than the average values of evergreen needleleaf forest.

For the validation dataset, the modeled GPP by both TL-RHM_sine and TL-RHM_sinesine can also track the seasonal variations of the measured GPP with a peak in summer for most of the ENF, DBF, and GRA sites and with a small significant seasonal change for the EBF site (**Supplementary Figures S8–S11**). The two types of TL-RHM can capture most of the low values of measured daily GPP during overcast days over the seasons but fail to capture the high values for the US_Ha1 site.

Although the GPPs simulated by the two types of TL-RHM show similar results for different vegetation types, the GPPs simulated by TL-RHM_sine are always greater than those by TL-RHM_sinesine for all sites, and the cause will be discussed in the discussion section. Moreover, the two types of TL-RHM behave differently in their simulation of daily GPP for different vegetation types. For the ENF, DBF, and GRA sites, the TL-RHM_sinesine performs slightly better than the TL-RHM_sine, while for the EBF sites, the TL-RHM_sine performs better.

## 4 Discussion

### 4.1 Comparison of the two types of daily TL-RHM

The difference between the two types of TL-RHM is the radiation function used in the daily integration with respect to time (Equations 4, 5). The maximum daily radiations simulated by a squared sine function used in TL-RHM_sinesine are always higher than those simulated by a sine function in TL-RHM_sine. This can be proven by deriving Equations 11, 12:


(11) $$\begin{array}{l} R_{\text{daily}}=\int_{0}^{\text{Daylength}}R_{\text{noon},\text{sine}}\sin\frac{\text{π}t}{\text{Daylength}}\text{ dt}\\ \text{ = -}R_{\text{noon},\text{sine}}\frac{\text{Daylength}}{\text{π}}\;{\text{cos}\frac{\text{πt}}{\text{Daylength}}|}_{0}^{\text{Daylength}}\\ \text{ =}\frac{2\times R_{\text{noon},\text{sine}}\times \text{Daylength}}{\text{π}} \end{array}$$

Then, the radiation at noon for the sine function, $R_{\text{noon},\text{sine}}=\frac{\text{π}R_{daily}}{2\times \text{Daylength}}$ .


(12) $$\begin{array}{l} R_{\text{daily}}=\int_{0}^{\text{Daylength}}R_{\text{noon},\text{sine}}\sin^{2}\frac{\text{π}t}{\text{Daylength}}\text{ dt}\\ \text{ = }R_{\text{noon},\text{squared-sine}}\frac{\text{Daylength}}{\text{π}}\;{\left[\frac{\text{π}t}{2\times \text{Daylength}}-\frac{1}{2}\sin\frac{2\text{π}t}{\text{Daylength}}\right]|}_{0}^{\text{Daylength}}\\ \text{ =}\frac{\;R_{\text{noon},\text{squared-sine}}\times \text{Daylength}}{2} \end{array}$$

**Figure 5:**
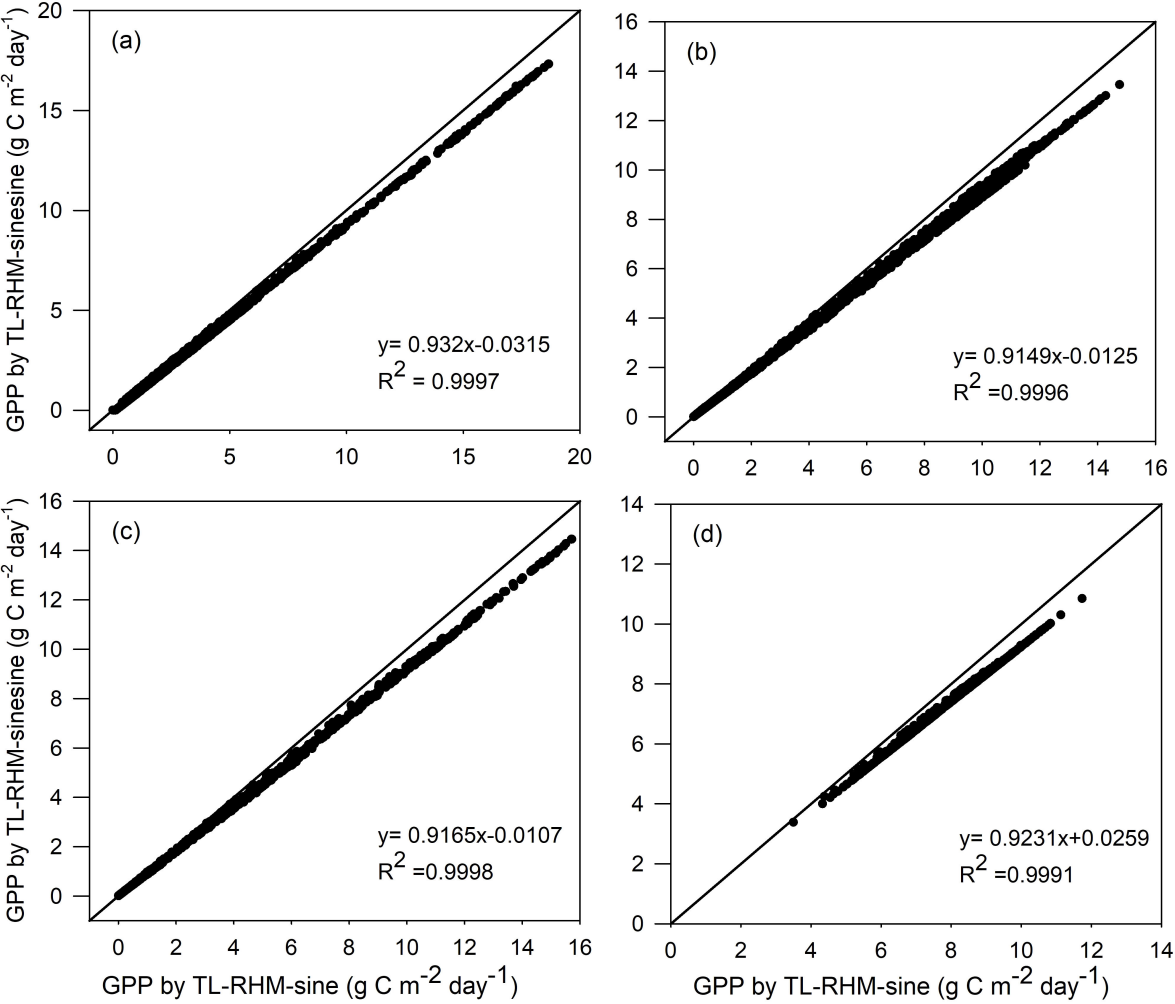
Comparisons of GPP simulated by the TL-RHM_sine and TL-RHM_sinesine for the sites of **(a)** evergreen coniferous forest, **(b)** broadleaf deciduous forest, **(c)** grassland, and **(d)** evergreen broadleaf forest. Diagonal lines are the 1:1 lines.

The radiation at noon for the squared sine function, $\;R_{\text{noon},\text{squared-sine}}=\frac{2\times R_{\text{daily}}}{\text{Daylength}}$ . Therefore, since 2 is greater than *PI/2*, we can draw a conclusion that *R*_noon,squared-sine_ is higher than *R*_noon,sine_, but except around the noon hours, the radiation simulated by a sine function is higher than that simulated by a squared sine function. Because GPPs at noon hours on clear days usually approach an asymptote, the increase in radiation at those hours does not produce a significant increase in the corresponding GPP, while the increase in radiation at morning or afternoon hours will result in an obvious increase in the corresponding GPP. Therefore, the modeled GPPs by TL-RHM_sine are always higher than those obtained by TL-RHM_sinesine, as shown in **Figure 5**. For ENF, DBF, GRA, and EBF, the *R* ^2^ values of the fitted linear equations between GPPs from TL-RHM_sine and TL-RHM_sinesine are close to 1, indicating that the GPPs simulated from the two types of TL-RHM are highly correlated with each other. The slopes of the fitted linear equations between GPPs are 0.93, 0.91, 0.92, and 0.92, respectively, for ENF, DBF, GRA, and EBF, with an average slope value of 0.92.

As to which type of TL-RHM performs better in GPP estimation, it depends on which radiation function (i.e., sine or squared sine) can accurately simulate the diurnal radiation variation at the study sites. In general, at high latitudes, solar radiation follows a squared sine function approximately, while it follows a sine function approximately at low latitudes. Therefore, TL-RHM_sine may be suitable for lower latitudes, while TL-RHM_sine may be appropriate for higher latitudes.

### 4.2 Limitations of daily TL-RHM

In the modified TL**-**RHM, we considered the effects of temperature and vegetation types on GPP. Although vapor pressure deficit was taken into account in the daily GPP estimation, it is only considered by an empirical function. Moreover, GPP is also affected by soil water content, but it is not included in daily TL-RHM. A possible solution is to introduce a soil water factor to the daily GPP model, similar to its application in LUE models. For large-scale applications, the water stress spectral index derived from remote sensing data can be used to downregulate daily GPP under conditions of deficient soil water content (Ceccato et al., 2002; Xiao et al., 2005). Finally, it should be noted that the influence of CO_2_ concentration on photosynthesis is also not considered, but it is important for long-term GPP simulation. In addition, since the daily TL-RHM is developed based on Baldocchi’s photosynthesis model, the uncertainties in Baldocchi’s model may be inherited by the daily TL-RHM.

In our study, the diurnal variations of radiation were assumed to follow a sine or a squared sine function. The diurnal radiation basically follows a sine or a squared sine function. However, for an individual day, the assumption may not be satisfied under some unstable weather conditions (e.g., concurrent sunny and cloudy days in the morning or afternoon), in which the estimated daily GPP may not be accurately calculated by TL-RHM, and in most cases, overestimation of daily GPP may occur.

## 5 Conclusions

In our study, two types of daily two-leaf GPP models were developed. The two models called TL-RHMs were constructed by integrating a temperature and vegetation type-adapted rectangular hyperbolic model with respect to time to make them suitable for different environmental conditions and vegetation types. Four eddy-covariance measurement sites with different vegetation types and an hourly process model (the BEPS model) were used to evaluate the performance of the two types of the daily GPP model. Comparison results showed that the newly developed TL-RHMs can not only track the seasonal trends of daily GPP but also capture the day-to-day variation of daily GPP. The RMSE values between daily GPPs measured and simulated by TL-RHM_sine are 2.261, 1.916, and 1.349 g C m^−2^ day^−1^ for the ENF, DBF, and GRA sites, respectively, and 2.001, 1.854, 1.214, and 1.230 g C m^−2^ day^−1^ by TL-RHM_sinesine for the ENF, DBF, GRA and EBF sites, respectively. The results mean that TL-RHMs can simulate a comparative daily GPP as accurately as a process model but with a lower complexity compared to the TL-LUE model, indicating the great potential of TL-RHMs for daily GPP simulation at large scales.

## Data Availability

Publicly available datasets were analyzed in this study. This data can be found here: The flux tower data sets used to calibrate and validate the new proposed model are acquired from the AmeriFlux website (http://ameriflux.ornl.gov), and the Canadian Carbon Program (CCP) website (http://fluxnet.ccrp.ec.gc.ca).
