# Supplementary material for: A two-leaf daily GPP model based on a rectangular hyperbolic model adjusted for air temperature and vegetation type
Source: Front Plant Sci. 2025 Mar 11;16:1555482. doi: 10.3389/fpls.2025.1555482 (PMC11933123; doi:10.3389/fpls.2025.1555482)
Supplement: Supplementary file 1 [file DataSheet1.docx]

Supplementary Material

# Supplementary Figures and Tables

**Supplementary Figure 1.** R^2^ distribution map of rectangular hyperbolic models for different temperatures and *V_cmax,25_.*

**Supplementary Figure 2.** Quantum yield (*α*) distribution map for different air temperatures and *V_cmax,25_*.

**Supplementary Figure 3.** Maximum photosynthetic rate (*P_m_*) distribution map for different temperatures and *V_cmax,25_.*

**Supplementary Figure 4.** Seasonal variations of measured GPP and simulated GPP using TL-RHM_sine model and TL-RHM_sinesine for calibration dataset of evergreen needleleaf forest.

**Supplementary Figure 5.** Seasonal variations of measured GPP and simulated GPP using TL-RHM_sine model and TL-RHM_sinesine for calibration dataset of deciduous broadleaf forest.

**Supplementary Figure 6.** Seasonal variations of measured GPP and simulated GPP using TL-RHM_sine model and TL-RHM_sinesine for calibration dataset of Grassland.

**Supplementary Figure 7.**  Seasonal variations of measured GPP and simulated GPP using TL-RHM_sine model and TL-RHM_sinesine for calibration dataset of evergreen broadleaf forest.

**Supplementary Figure 8.**  Seasonal variations of measured GPP and simulated GPP using TL-RHM_sine model and TL-RHM_sinesine for validation dataset of evergreen needleleaf forest.

**Supplementary Figure 9.** Seasonal variations of measured GPP and simulated GPP using TL-RHM_sine model and TL-RHM_sinesine for validation dataset of deciduous broadleaf forest.

**Supplementary Figure 10.** Seasonal variations of measured GPP and simulated GPP using TL-RHM_sine model and TL-RHM_sinesine for validation dataset of Grassland.

**Supplementary Figure 11.**  Seasonal variations of measured GPP and simulated GPP using TL-RHM_sine model and TL-RHM_sinesine for validation dataset of evergreen broadleaf forest.

**Supplementary Table 1.** The inputs of daily TL-RHM.

## Supplementary Figures


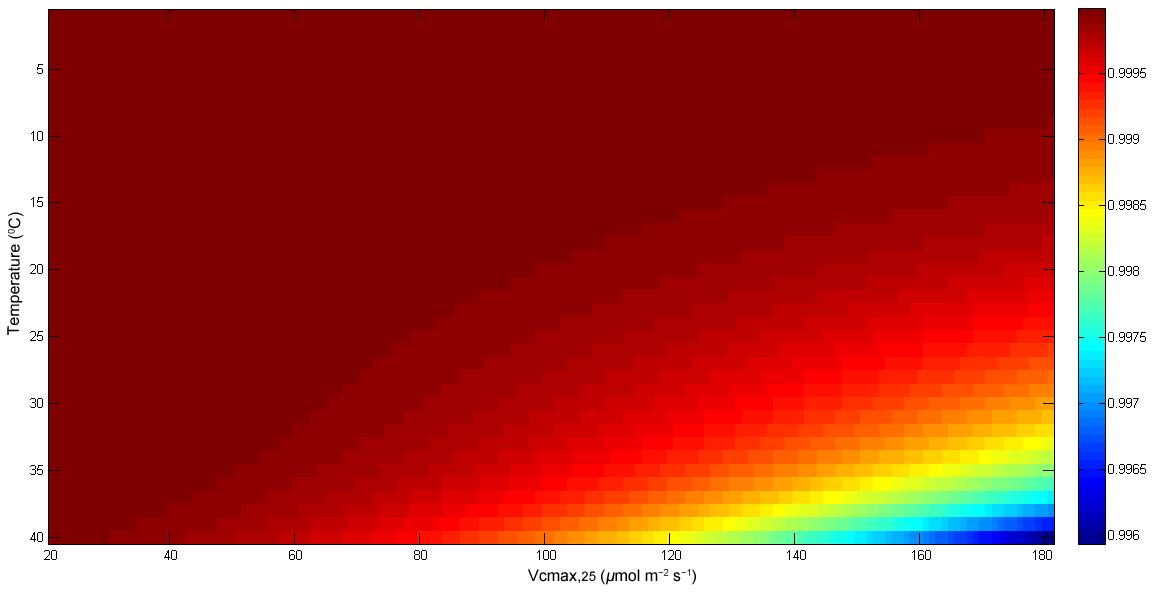


**Supplementary Figure 1.** R^2^ distribution map of rectangular hyperbolic models for different temperatures and *V_cmax,25_.*


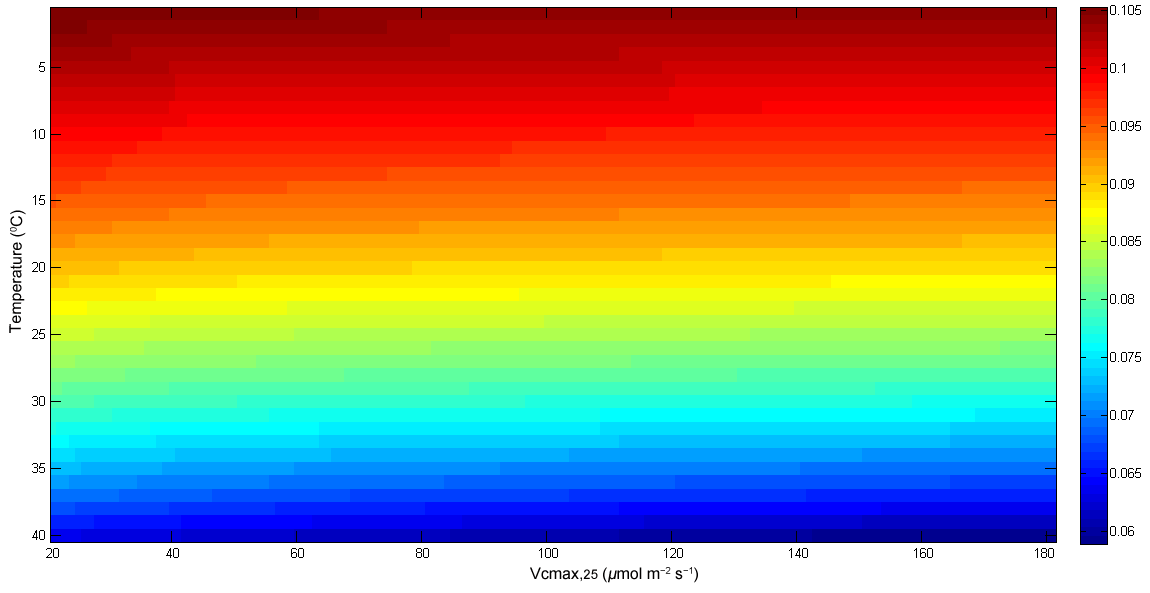


**Supplementary Figure 2.** Quantum yield (*α*) distribution map for different air temperatures and *V_cmax,25_*.


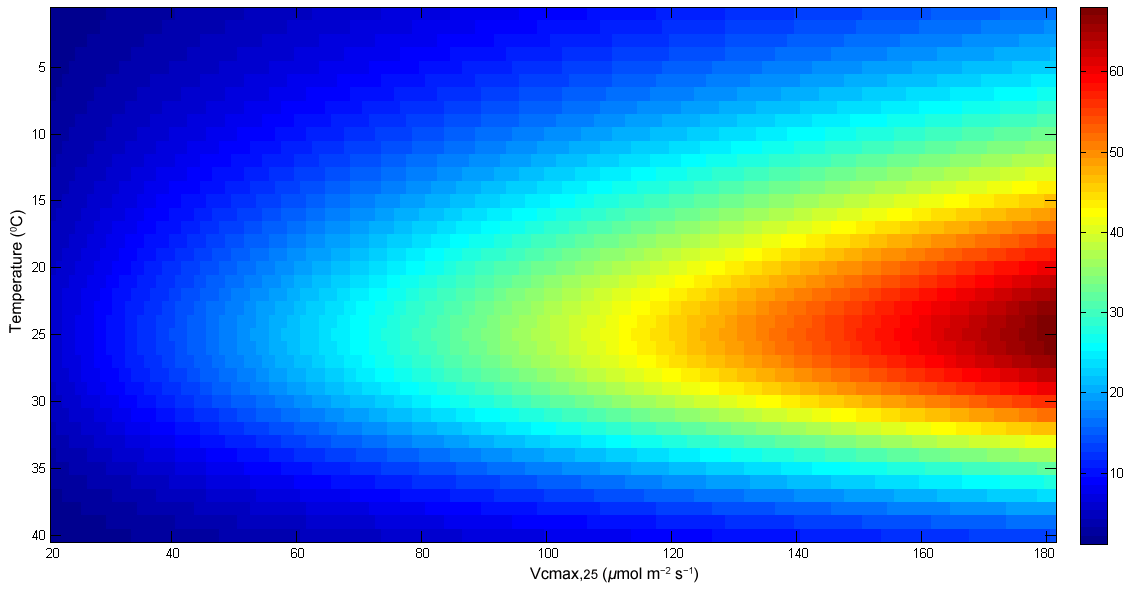
**Supplementary Figure 3.** Maximum photosynthetic rate (*P_m_*) distribution map for different temperatures and *V_cmax,25_.*

**Supplementary Figure 4.** Seasonal variations of measured GPP and simulated GPP using TL-RHM_sine model and TL-RHM_sinesine for calibration dataset of evergreen needleleaf forest.

**Supplementary Figure 5.** Seasonal variations of measured GPP and simulated GPP using TL-RHM_sine model and TL-RHM_sinesine for calibration dataset of deciduous broadleaf forest.

**Supplementary Figure 6.** Seasonal variations of measured GPP and simulated GPP using TL-RHM_sine model and TL-RHM_sinesine for calibration dataset of Grassland.

**Supplementary Figure 7.**  Seasonal variations of measured GPP and simulated GPP using TL-RHM_sine model and TL-RHM_sinesine for calibration dataset of evergreen broadleaf forest.

**Supplementary Figure 8.**  Seasonal variations of measured GPP and simulated GPP using TL-RHM_sine model and TL-RHM_sinesine for validation dataset of evergreen needleleaf forest.

**Supplementary Figure 9.** Seasonal variations of measured GPP and simulated GPP using TL-RHM_sine model and TL-RHM_sinesine for validation dataset of deciduous broadleaf forest.

**Supplementary Figure 10.** Seasonal variations of measured GPP and simulated GPP using TL-RHM_sine model and TL-RHM_sinesine for validation dataset of Grassland.

**Supplementary Figure 11.**  Seasonal variations of measured GPP and simulated GPP using TL-RHM_sine model and TL-RHM_sinesine for validation dataset of evergreen broadleaf forest.

## Supplementary Tables

**Supplementary Table 1.** The inputs of daily TL-RHM.

| No. | Inputs | Explanation |
| --- | --- | --- |
| 1 | Radiation | Daily meteorological data |
| 2 | Minimum air temperature |  |
| 3 | Maximum air temperature |  |
| 4 | Relative humidity |  |
| 5 | LAI | Leaf area index |
| 6 | Land cover | For determination of vegetation type |
| 7 | Longitude | For calculation of daylength |
| 8 | Latitude |  |
| 9 | Quantum yield (α) matrix | For determination of Quantum yield (α) by temperature and Vcmax,25. |
| 10 | Maximum photosynthetic rate (Pm) matrix | For determination of photosynthetic rate (Pm) by temperature and Vcmax,25. |
